# Supplementary material for: The Plasmodium berghei RC strain is highly diverged and harbors putatively novel drug resistance variants
Source: PeerJ. 2017 Oct 5;5:e3766. doi: 10.7717/peerj.3766 (PMC5632537; doi:10.7717/peerj.3766)
Supplement: Supplemental Information 1 [file peerj-05-3766-s001.docx]

| **Primer**  **ID** | **Primer name** | **Gene target** | **Sequence 5′ - 3′** |
| --- | --- | --- | --- |
| 238 | *Ubp1*-1F | PBANKA_0208800 | GTTCCGATTCCAAAGACAATG |
| 239 | *Ubp1*-1R | PBANKA_0208800 | TAACGGATCTCTTTTTTCCATCA |
| 240 | *Ubp1*-2F | PBANKA_0208800 | GTGAATAATAAGAAAGATATACGAG |
| 241 | *Ubp1*-2R | PBANKA_0208800 | GGAGTATGAATATATTGCTTGCT |
| 242 | *Ubp1*-3F | PBANKA_0208800 | TATTCCCCTAGTGAAGAAAAAAAAA |
| 243 | *Ubp1*-3R | PBANKA_0208800 | TTTGGGGTCTAGAACAACGA |
| 244 | *Ubp1*-4F | PBANKA_0208800 | CTGTACACGCCAAGGAAAAT |
| 245 | *Ubp1*-4R | PBANKA_0208800 | CTTTCAATTGATGTGCTATGAG |
| 246 | *Ubp1*-5F | PBANKA_0208800 | AACTATTTGCAACCAAACTC GA |
| 247 | *Ubp1*-5R | PBANKA_0208800 | GACATTTGTTTGAATACTTTTACGA |
| 252 | *gcs*-2F | PBANKA_0819800 | CAAAAATGTGGAGCGAAGAG |
| 14 | *gcs*-2R | PBANKA_0819800 | ctactgTCTAGActatgcactgaattgatacaac |
| 253 | *Mdr1*-1F | PBANKA_1237800 | AAGAAATCTACCGTTGAGCTG |
| 254 | *Mdr1*-1R | PBANKA_1237800 | GAAGCCACTACCGTAATTCA |
| 255 | *Mdr1*-2F | PBANKA_1237800 | TGGCAGGTGGTTTATATCCA |
| 256 | *Mdr1*-2R | PBANKA_1237800 | CTCCTGCTATTATTGGGCAAA |
| 266 | *crt*-1F | PBANKA_12195000 | TGATCGCTATAAAGAATTGGAC |
| 267 | *crt*-1R | PBANKA_12195000 | GAATATTTCCCGTTGTTCTTGT |
| 259 | *crt*-2F | PBANKA_12195000 | GGTAGGGCATAATTTTGCAC |
| 260 | *crt*-2R | PBANKA_12195000 | TTGTTATGGCTGGTCCTTGT |
