# Supplementary figures and images for: The Plasmodium berghei RC strain is highly diverged and harbors putatively novel drug resistance variants
Source: PeerJ. 2017 Oct 5;5:e3766. doi: 10.7717/peerj.3766 (PMC5632537; doi:10.7717/peerj.3766)

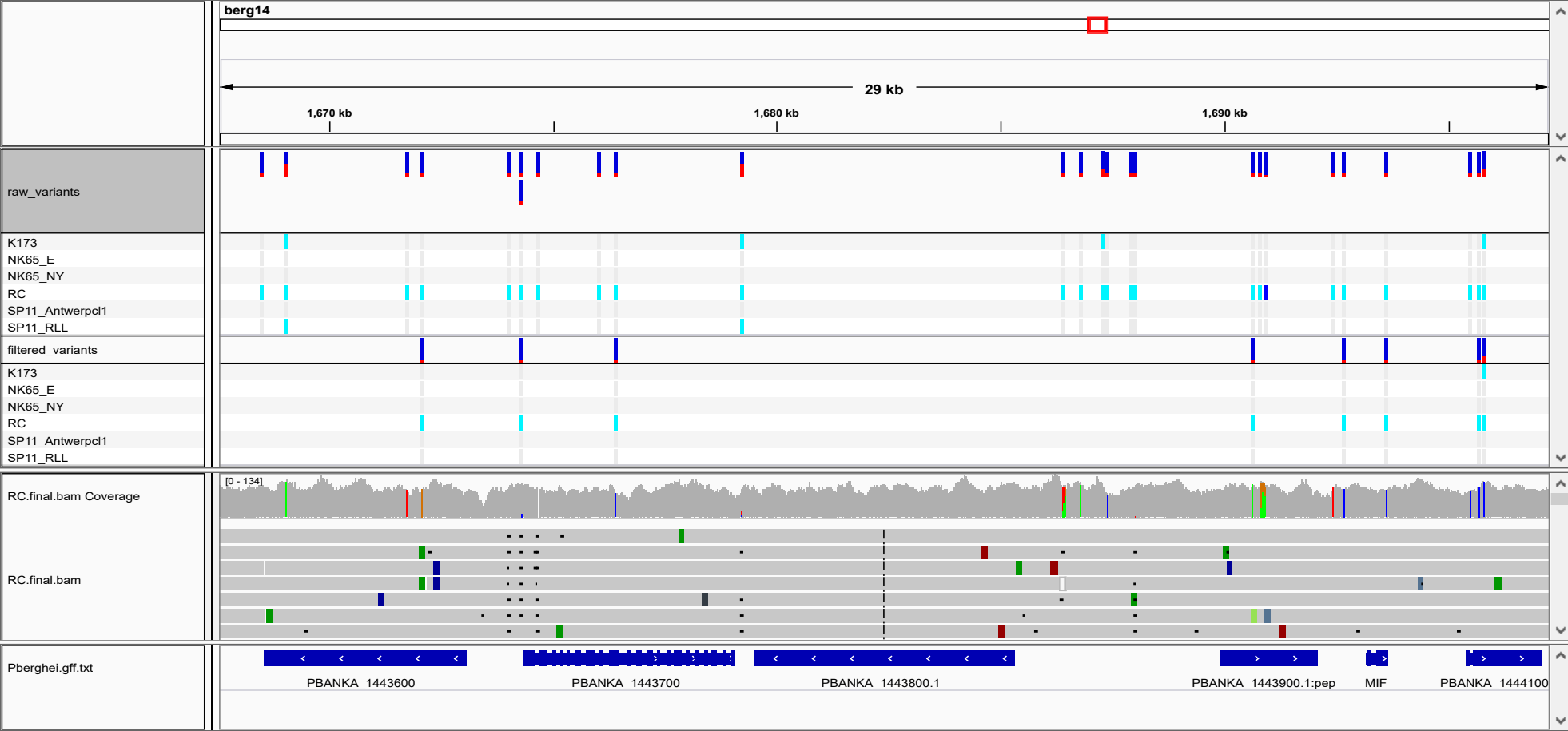

Supplement: Supplemental Information 6 — Integrative Genomics Viewer screenshot showing P. berghei RC reads mapped to chromosome 14 (berg14) in the vicinity of PBANKA_1443800.1 (multidrug resistance associated protein, MRP). Mapped reads from P. berghei RC are shown in the tracks labeled RC.final.bam.Coverage and RC.final.bam. Genomic variants among the six P. berghei strains before and after filtering are shown in the tracks labeled raw_variants and filtered_variants, respectively. [file peerj-05-3766-s006.pdf]
